# Paediatric dermatofibrosarcoma protuberan—a case report in an Afro-Caribbean boy

**DOI:** 10.1093/jscr/rjae062

**Published:** 2024-02-13

**Authors:** Carlos Neblett, Kenneth Appiah, Javier Jones, Tahjeme Lawrence, Shanna Kay Dawkins, Graeme Crookendale, Rory Thompson

**Affiliations:** Division of Plastic & Reconstructive Surgery, Department of Surgery, Bustamante Hospital for Children, 5 Arthur Wint Drive, Kingston 5, JMAAW04, Jamaica; Division of Plastic & Reconstructive Surgery, Department of Surgery, Bustamante Hospital for Children, 5 Arthur Wint Drive, Kingston 5, JMAAW04, Jamaica; Division of Plastic & Reconstructive Surgery, Department of Surgery, Bustamante Hospital for Children, 5 Arthur Wint Drive, Kingston 5, JMAAW04, Jamaica; Division of Plastic & Reconstructive Surgery, Department of Surgery, Bustamante Hospital for Children, 5 Arthur Wint Drive, Kingston 5, JMAAW04, Jamaica; Division of Plastic & Reconstructive Surgery, Department of Surgery, Bustamante Hospital for Children, 5 Arthur Wint Drive, Kingston 5, JMAAW04, Jamaica; Division of Plastic & Reconstructive Surgery, Department of Surgery, Bustamante Hospital for Children, 5 Arthur Wint Drive, Kingston 5, JMAAW04, Jamaica; Department of Pathology, University Hospital of the West Indies, Mona, Kingston 7, JMAAW15, Jamaica

**Keywords:** Boy, dermatofibrosarcoma protuberans, trunk, Afro-Caribbean

## Abstract

Dermatofibrosarcoma protuberans is a rare low-grade sarcoma, which rarely metastasizes, but it is locally aggressive with a propensity to recur. It usually affects persons of African descent and is extremely rare in childhood with a favourable prognosis. We present a case of paediatric dermatofibrosarcoma protuberans to the midline of the lower back of a 9-year-old Afro-Caribbean boy who was biopsied with a 2-mm margin. After histological confirmation, a 4-cm margin was then performed. Surveillance for recurrence, though none has been seen thus far after 6-month follow-up, will be done for at least 5 years and possibly longer, given this is the first case of this nature ever seen in our institution and the Caribbean region.

## Introduction

Dermatofibrosarcoma protuberans (DFSP) is an infiltrative dermal and subcutaneous low-to-intermediate grade spindle cell neoplasm with limited metastatic but high local recurrence rate propensity [1]. The incidence of this rare tumour is approximately 1 in 1 000 000 in the paediatric population [2]. Surgical management employing Mohs micrographic surgery (MMS) and wide local resection (WLE) using 2–4-cm margins have been recommended. Difficulty in making the diagnosis in this age group and late presentation with large lesions commonly make achieving clear margins challenging [1–4]. We present a case of paediatric DFSP, the first such published case in the Caribbean after reviewing the literature.

## Case presentation

A 9-year-old boy of African descent presented with a swelling to the lower back, which evolved from a reported congenital nonindurated patch to a noticeable nodule at the age of 6, with progressive enlargement over the following 30 months and rapid growth over the last 6 months, prompting initial medical attention being sought. There were no constitutional nor other symptoms throughout this course. Medical history was unremarkable.

**Figure 1:**
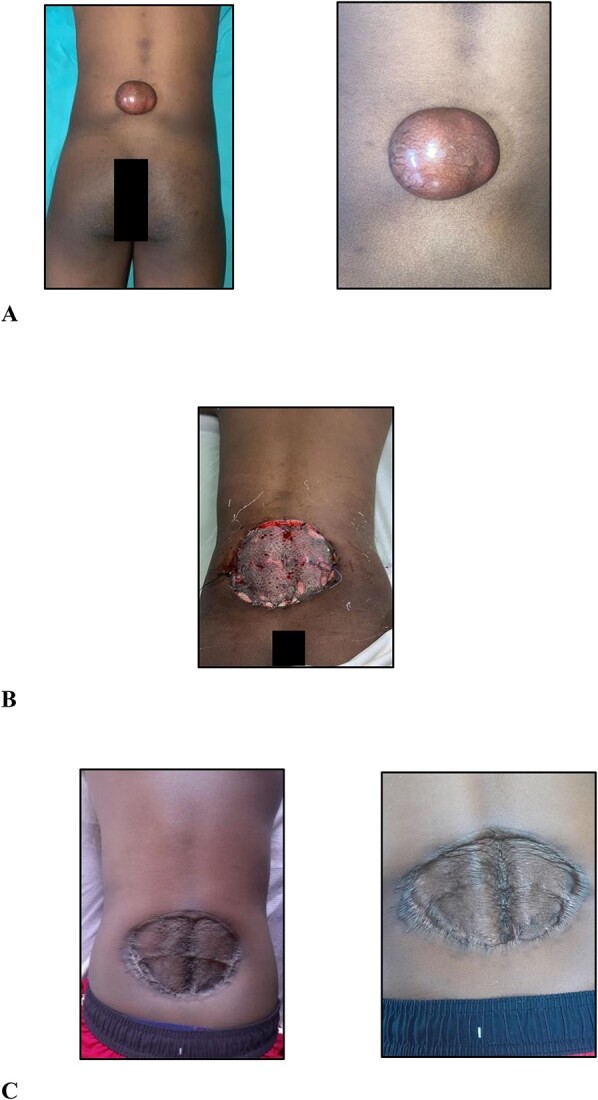
(A) Clinical photographs of the 6.0 × 5.0 × 3.0-cm hyperpigmented, spherical, firm, smooth, mobile nodule to the midline of the lower back 5.0 cm cranial to the natal cleft; (B) clinical photographs of the skin-grafted lower back at 3 days after surgery; (C) clinical photographs of the skin-grafted lower back at 6-month follow-up after surgery.

On examination, there was a 6.0 × 5.0 × 3.0-cm hyperpigmented, spherical, firm, smooth, mobile, nontender nodule to the midline of the lower back 5.0 cm cranial to the natal cleft (Fig. 1A). The mass involved the subcutaneous plane and overlying skin but not underlying musculature with no groin lymphadenopathy.

**Figure 2:**
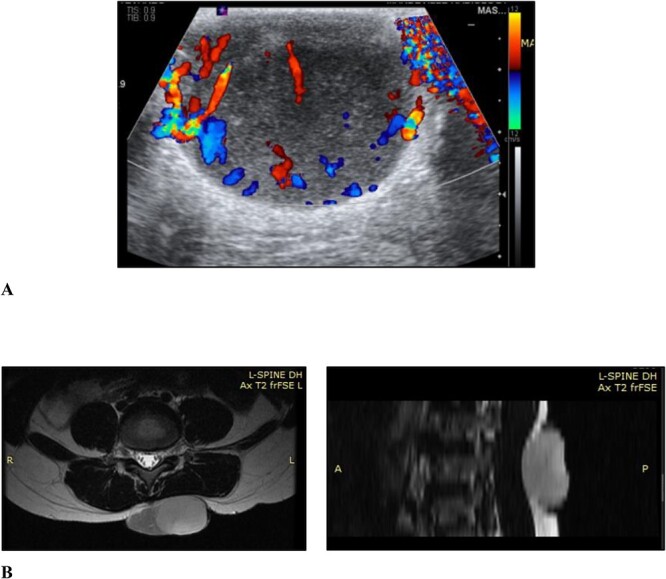
(A) Ultrasound scan of the lesion to the lower back with doppler showing vascular flow; (B) MRI scan of the lesion to the back showing it extending to the level of the erector spinae fascia but not crossing it and no tract to the spinal canal, in the axial and sagittal planes, respectively.

Ultrasound examination revealed a well-circumscribed heterogenous lesion measuring 4.8 × 4.0 × 2.9 cm, with areas of increased flow on doppler imaging but no vascular pedicle, phleboliths nor calcifications (Fig. 2). Magnetic resonance imaging (MRI) examination showed a well-defined 5.4 × 4.6 × 2.7-cm lesion, centred in the subcutaneous tissues of the back, extending to the erector spinae fascia but not crossing it and no tract to the spinal canal (Fig. 2).

Haematological test results revealed a normal leukocyte count of 4.78 × 10^9^/l (normal range is 4–15 × 10^9^/l).

**Figure 3:**
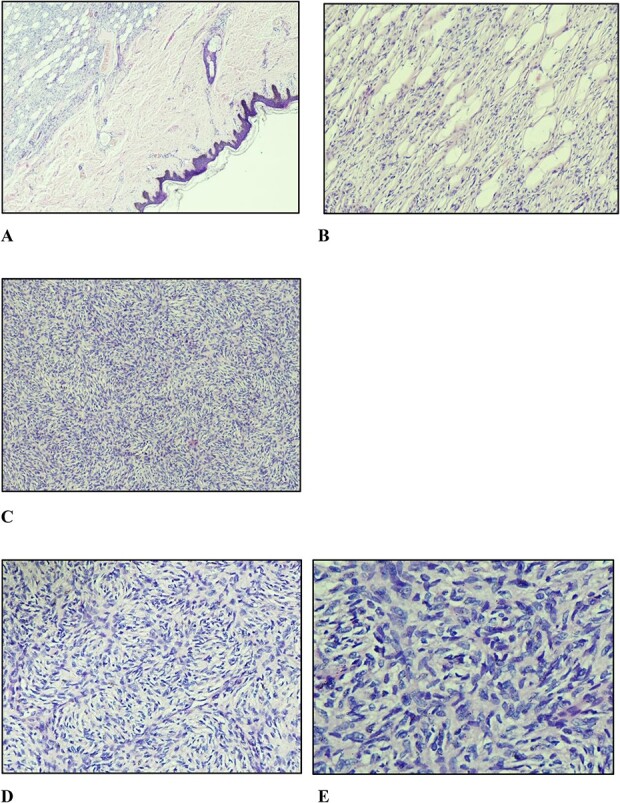
(A) Nodular vaguely circumscribed spindle cell proliferation centred in the subcutis with intact overlying skin (×40); (B) spindle cell proliferation interdigitating the adipose tissue resulting in a honeycomb pattern (×100); (C and D) spindle cells showing the typical storiform pattern of growth (×100 and ×200); (E) slender fibroblastic tumour cells displaying elongated wavy nuclei with mild to minimal atypia.

A marginal (2 mm circumferentially and deep) excision biopsy was performed. Histopathological findings were: a circumscribed, markedly cellular, nodular lesion composed of spindle cells disposed in a storiform pattern, which infiltrate the fibrous septa of the subcutis in the usual honeycomb pattern with mitoses of 5 per 10 high power fields, with uninvolved resection margins, consistent with but not limited to, DFSP (Fig. 3). Immunohistochemistry studies were positive for CD34, while they were negative for STAT6 and CD163.

After multidisciplinary team meeting, decision was made for surgical management using 4.0-cm circumferential margins, resection of the corresponding deep fascia overlying the erector spinae muscles, and no adjuvant radiation therapy. The defect was resurfaced with a meshed split thickness skin graft (Fig. 1B). At 6-month postoperative stage (Fig. 1C), there are no signs of recurrence. Close outpatient surveillance will continue over the next 5 years and beyond.

## Discussion

DFSPs, coined by Hoffman, described by Darier and Ferrand, are progressive, recurring dermatofibroma, hypertrophic morphea, and sarcomatous tumours resembling keloid and fibrosarcoma of the skin. The pathogenesis is unknown, and tumour cells carry abnormal chromosomes within—t(17, 22) (q22; q13)—resulting in the fusion gene collagen type 1α 1 gene—platelet derived growth factor β, which encodes a protein that causes tumour growth [1].

As in adults, histological and immunohistochemical features are similar in children, however, only 6% of these tumours are seen in the paediatric population [5]. Clinically, they occur on the trunk (40%–60%), proximal limbs (20%–30%), and the head and neck region (10%–16%) in the adult population [6]. In children, they typically appear on the legs and acral regions, though the congenital forms follow the adult distribution as in this case [1, 2]. The incidence in those of African descent is twice that of Caucasians with no sex predilection unlike in the adult population, where it is more common in women [7]. DFSPs have a low chance of metastasis, but it is locally aggressive [1].

### Histopathological appraisal of DFSP

Histologically, DFSPs exhibit small, elongated cells in a storiform pattern infiltrating the subcutaneous fat with a honeycomb pattern along with mitosis no more than five per high power fields, all present in this case. These features aid in distinguishing it from other entities but not definitively so. Therefore, immunohistochemistry studies; staining positively for CD34 and P75, negative for stromelysin, CD163 and STAT6, along with fluorescence *in situ* hybridization (FISH) for the platelet-derived growth factor-b rearrangement confirmation, are useful ancillary tests to confirm diagnosis [5]. In this report, the lesion was positive for CD34, while it was negative for STAT6 and CD163. Stromelysin, P75, and FISH assays were unavailable in our country.

### Principles of DFSP management

Given its rarity in children and diagnostic difficulty, delayed presentation is common. Patients present with relatively large lesions that complicate surgery since microscopically negative resection margins, the standard of care to achieve cure, and limited recurrence are difficult to achieve and recurrence rates are as high 60% [2]. Accurate and early diagnosis, employing immunohistochemistry analysis and FISH applications following histological assessment, is critical to guide an appropriate management in children. Tumour-negative margins realized by either WLE (2–4 cm), with the 4-cm excision margins being employed in this case, or MMS, also unavailable in our country, have recurrence rates as low as 9% and 3%, respectively [8].

In surgical unresectable, recurrent, or metastatic cases in the adult population targeted therapy using the tyrosine kinase inhibitor; imatinib mesylate has been employed, however, no standard clinical guidance exists for the paediatric patient [1, 2, 5]. Adjuvant radiation therapy, utilized when surgical margins are positive or close and reresection not feasible, has not been extensively studied in children. One study employed adjuvant radiation to the extremities, but limitations affected recurrence rates reporting [2]. Overall prognosis of paediatric DFSP is favourable with 15-year and 30-year overall survival rates being 98% and 97%, respectively, similar to the 15-year rates in adults of 97.2% [1, 2, 7, 8]. Clinical follow-up is required every 6–12 months, particularly in the first 3–5 years after surgery, inclusive of surgical scar and regional lymph node palpation [2].
